# Regulation of Insulin Resistance and Adiponectin Signaling in Adipose Tissue by Liver X Receptor Activation Highlights a Cross-Talk with PPARγ

**DOI:** 10.1371/journal.pone.0101269

**Published:** 2014-06-27

**Authors:** Fenping Zheng, Saifei Zhang, Weina Lu, Fang Wu, Xueyao Yin, Dan Yu, Qianqian Pan, Hong Li

**Affiliations:** 1 Department of Endocrinology, Sir Run Run Shaw Hospital Affiliated with School of Zhejiang University, Hangzhou, Zhejiang, P. R. China; 2 Biomedical Research Center and Key Laboratory of Biotherapy of Zhejiang Province, Sir Run Run Shaw Hospital Affiliated with School of Zhejiang University, Hangzhou, Zhejiang, P.R. China; University of Barcelona, Spain

## Abstract

Liver X receptors (LXRs) have been recognized as a promising therapeutic target for atherosclerosis; however, their role in insulin sensitivity is controversial. Adiponectin plays a unique role in maintaining insulin sensitivity. Currently, no systematic experiments elucidating the role of LXR activation in insulin function based on adiponectin signaling have been reported. Here, we investigated the role of LXR activation in insulin resistance based on adiponectin signaling, and possible mechanisms. C57BL/6 mice maintained on a regular chow received the LXR agonist, T0901317 (30 mg/kg.d) for 3 weeks by intraperitoneal injection, and differentiated 3T3-L1 adipocytes were treated with T0901317 or GW3965. T0901317 treatment induced significant insulin resistance in C57BL/6 mice. It decreased adiponectin gene transcription in epididymal fat, as well as serum adiponectin levels. Activity of AMPK, a key mediator of adiponectin signaling, was also decreased, resulting in decreased Glut-4 membrane translocation in epididymal fat. In contrast, adiponectin activity was not changed in the liver of T0901317 treated mice. *In vitro*, both T0901317 and GW3965 decreased adiponectin expression in adipocytes in a dose-dependent manner, an effect which was diminished by LXRα silencing. ChIP-qPCR studies demonstrated that T0901317 decreased the binding of PPARγ to the PPAR-responsive element (PPRE) of the adiponectin promoter in a dose-dependent manner. Furthermore, T0901317 exerted an antagonistic effect on the expression of adiponectin in adipocytes co-treated with 3 µM Pioglitazone. In luciferase reporter gene assays, T0901317 dose-dependently inhibited PPRE-Luc activity in HEK293 cells co-transfected with LXRα and PPARγ. These results suggest that LXR activation induces insulin resistance with decreased adiponectin signaling in epididymal fat, probably due to negative regulation of PPARγ signaling. These findings indicate that the potential of LXR activation as a therapeutic target for atherosclerosis may be limited by the possibility of exacerbating insulin resistance-related disease.

## Introduction

Liver X receptors (LXRs), including α and β isoforms, are transcription factors belonging to the nuclear receptor superfamily. Animal studies have shown positive effects of LXR activation on cholesterol reverse transport and anti-inflammation in macrophages; thus, LXR has been identified as a promising pharmacological target for the management of atherosclerosis [1]–[3]. However, these beneficial effects are accompanied by severe hyperlipidemia and hepatic steatosis [4]–[5]. A possible involvement of dysregulated LXR signaling in glucose metabolism and insulin resistance has been proposed in several independent studies; however, the results are controversial. For example, previous studies by Cao et al. [6] and Laffitte et al. [7] show that LXR activation improves glucose tolerance in insulin-resistant Zucker (fa/fa) rats and in a murine model of diet-induced obesity and insulin resistance. In these studies, positive outcomes are primarily mediated by inhibition of gluconeogenesis and limitation of glucose output from the liver; however, recent studies using LXR-knockout mice have demonstrated opposing results [8]–[9]. Furthermore, Meng et al. [10] has shown decreased β cell glucose sensitivity and insulin secretion in response to LXR activation *in vitro* and deterioration of glucose tolerance *in* *vivo* manifested as suppressed insulin secretion in response to glucose injection. Another study has also demonstrated that LXR activation downregulated insulin-stimulated glucose uptake in human adipocytes from overweight individuals, which could be due to transcriptional suppression of several insulin signaling genes [11]. Over the last decade, accumulating evidence suggests that adipose tissue and secreted adipokines play a unique role in maintaining whole-body insulin sensitivity [12]–[13]. Both LXRα and β are expressed in fat tissue and the former is increased during adipogenesis and adipocyte differentiation, indicating that LXRα might play a more important role in adipose function [14]–[15]; however, the role of LXR activation in insulin function in fat tissue remains to be clarified.

Adiponectin has been identified as one of the most abundant adipose-specific adipokines and plays a critical role in the maintenance of insulin sensitivity and metabolic homeostasis. Decreased plasma adiponectin has been found in patients affected by obesity, type 2 diabetes and other insulin-resistant states, despite increasing amounts of fat tissue [16]. Numerous studies in animal models have consistently shown that adiponectin mediates anti-diabetic effects via insulin-sensitizing or insulin-mimetic effects in various tissues [17]–[18]. Adiponectin acts locally and distally through autocrine, paracrine and endocrine effects after secretion from adipose tissue. It exerts its insulin-sensitizing activity through the receptors AdipoR1 and AdipoR2, which are broadly expressed in adipose tissue, muscle and liver, with predominant roles of AdipoR1 in muscle and AdipoR2 in liver. AMPK and PPARα signaling are the main post-receptor events mediating the action of adiponectin in different organs [19]. The transcription of adiponectin is dominantly regulated by peroxisome proliferator-activated receptor-gamma (PPARγ), a transcription factor belonging to the nuclear receptor family. A functional PPAR-responsive element (PPRE) has been identified in the murine and human adiponectin promoters. The PPARγ/retinoid X receptor (RXR) heterodimer binds directly to the PPRE and increases adiponectin promoter activity in adipocytes [20].

Currently, no systematic experiments elucidating the role of LXR activation in insulin function based on adiponectin signaling have been reported, although this may represent its most important role in the whole-body insulin sensitivity. Furthermore, the induction of adiponectin is dominantly regulated by PPARγ at the transcriptional level in adipose tissue. It has been confirmed in the liver that LXRs shares its heterodimerization partner RXR with PPARs and the LXR-responsive element (LXRE) is present in the PPARγ promoter [14], [21]. These factors suggest that LXR activation could also affect PPARγ signaling, leading to unexpected consequences when administering LXR activators *in vivo* and *in vitro*, and thus influencing adiponectin signaling activity.

In this study, we examined the effects of LXR activation on insulin resistance and adiponectin activity in epididymal (EP) fat tissue and liver to further elucidate a possible link of LXR activation with insulin resistance. Moreover, the mechanism by which LXR activation regulates adiponectin expression was investigated taking into account the signaling interactions between different nuclear receptors.

## Materials and Methods

### Reagents

T0901317 was purchased from Cayman Chemical Company (Ann Arbor, Michigan, USA). Pioglitazone and GW3965 were purchased from Sigma-Aldrich (Saint Louis, Missouri, USA). In animal experiments, T0901317 was solubilized in a vehicle containing 3% dimethyl sulfide (DMSO) in PBS and administered by intraperitoneal injection at a dose of 30 mg/kg body weight. In cellular experiments, T0901317 and GW3965 were prepared in a solution of 1∶1 DMSO: PBS at a concentration of 1 mM, and Pioglitazone was solubilized in DMSO at a concentration of 20 mM for further dilution with cell medium.

### Animals

All animal care procedures and methods were approved by the Animal Care Committee of Zhejiang University (China). C57BL/6 mice (aged 11 weeks) were purchased from Slack Experimental Animal Center of Chinese Academy of Sciences (Shanghai, China) and were fed a regular chow diet (carbohydrate, 63.92%; protein, 26.18%; fat, 9.9%). A cohort of 30 mice were housed singly and maintained on a 12-h light-dark cycle. After 1 week of habituation to intraperitoneal (i.p.) injection of saline on alternate days, mice were treated for 3 weeks with 30 mg/kg T0901317 per day (*n = *16) or the vehicle (3% DMSO in PBS, *n = *14) by i.p injection. This dose has been shown to be effective in the treatment of atherosclerosis [22]. The body weight was recorded once a week.

### In vivo glucose homeostasis assays

After 3 weeks of treatment, intraperitoneal glucose tolerance tests (IPGTT) and intraperitoneal insulin tolerance tests (ITT) were carried out. Half of the mice (*n* = 7–8 per group) were injected i.p. with glucose (1.5 g/kg body weight) following an overnight fast and blood glucose levels were measured from tail blood using One Touch Ultra glucose strips (LifeScan) at 0 (basal), 15, 30, 60 and 120 min. Tail blood was also sampled at each time-point for insulin measurements. The remaining mice (*n* = 7–8 per group) were subjected to ITT. Mice were fasted for 4 h prior to i.p. injection of insulin at a dose of 0.5 IU/kg body weight. Blood samples were collected from the tail at 0, 15, 30, 60 and 120 min, and glucose levels were measured immediately by One Touch Ultra glucose strips (LifeScan).

After these tests, mice were fasted for 16 h and injected i.p. with saline (n = 7–8 per group) or insulin (10 U/kg body weight, *n* = 7–8 per group) and sacrificed 3 min later by cervical dislocation following ether anesthesia. Blood samples were obtained and serum was collected and stored at −80°C immediately. Liver, epididymal and inguinal fat pads were carefully excised and weighed. After rinsing in pre-cooled PBS, part of the tissue was placed in storage tubes in a dry ice bath until the end of experiment, and then stored at −80°C for later protein, RNA extraction and preparation of frozen sections, while the other part was fixed in 4% formaldehyde.

### Blood sample assays

Serum insulin concentrations were determined using the insulin (mouse) EIA kit (Millipore, Massachusetts, USA). Adiponectin levels in the fasted serum were measured with the adiponectin (murine) EIA kit (Millipore). Free fatty acid, leptin and triglycerides levels in the fasted serum were measured with the FFA assay kit (Cayman Chemical), mouse leptin ELISA kit (Millipore) and mouse TG quantification colormetric kit (Bivision). All kits were used according to the manufacturer’s protocols.

### Histological analysis of adipose tissue

Tissues fixed in 4% formaldehyde were then embedded in OCT compound and cut into sections (thickness, 4 µm) according to a standard protocol. The sections were stained with hematoxylin and eosin (H&E) and examined under a light microscope. For immunohistochemical staining of membrane-bound Glut-4, sections (thickness, 4 µm) were prepared from representative blocks of paraffin-embedded tissues, dewaxed, and rehydrated. Sections were then blocked with 3% H_2_O_2_ in PBS for 10 min at room temperature. To block non-specific binding, sections were incubated in 10% goat serum for 1 h. Sections were then incubated with rabbit anti-Glut4 antibody (Abcam, Cambridge, UK) at a dilution of 1∶4,000 in 10% goat serum overnight at 4°C. Goat serum replaced the primary antibody in negative controls. After several washes with PBS, sections were incubated with the Cy2-conjugated goat anti-rabbit secondary antibody (H&L, Alexa Fluor 488; Abcam) at a 1∶1,000 dilution for 1 h at room temperature. Sections were then counterstained with DAPI (4′6 diamidi-no-2-phenylindole, for nuclei staining), cover-slipped and examined under a fluorescence microscope.

### Quantitative real-time RT-PCR

Total RNA was isolated using TRIzol (Invitrogen, Grand Island, NY, USA) and reverse-transcribed with random hexamers by using TaqMan reverse-transcription reagents kit (Applied Biosystems Inc., San Francisco, CA, USA) according to the manufacturer’s protocol. Real-time PCR was performed using the 7500 real-time PCR system (ABI Applied Biosystems) and SYBR Green qPCR Kit (TaKaRa). Relative expression was normalized to that of GAPDH as an internal control for quantification of individual mRNA species and calculated using the formula 2^(−ΔΔCt)^. Primer sets included: (GAPDH) Forward: 5-TGCAC CACCA ACTGC TTAG-3, (GAPDH) Reverse: 5-GGATG CAGGG ATGAT GTTC-3; (Adiponectin) Forward: 5′-GTCAG TGGAT CTGAC GACAC CAA-3′; (Adiponectin) Reverse: 5′-ATGCC TGCCA TCCAA CCTG-3′; (AdipoR1) Forward: 5′-TGCCC TCCTT TCGGG CTTGC-3′; (AdipoR1) Reverse: 5′-GCCTT GACAA AGCCC TCAGC GATAG-3′; (AdipoR2) Forward: 5′-TCTTC CTGTG CCTGG GGATC TT-3′; (AdipoR2) Reverse: 5′-CCCGA TACTG AGGGG TGGCA AA-3′; (Glut-4) Forward: 5′-GTAAC TTCAT TGTCG GCATG G-3′; (Glut-4) Reverse: 5′-AGCTG AGATC TGGTC AAACG-3′; (LXRα) Forward: 5′-GAGAA GCTGG TGGCT GCCCA-3′; (LXRα) Reverse: 5′-AGCTG TAGGA AGCCA GGGAG-3′; (PPARγ) Forward: 5′-TGTCG GTTTC AGAAG TGCCT TG-3′; (PPARγ) Reverse: 5′-TTCAG CTGGT CGATA TCACT GGAG-3′.

### Western blotting

Equal amounts of protein (50 µg) denatured by boiling were separated by 10% SDS-polyacrylamide gel electrophoresis, transferred to an Immun-Blot PVDF membranes (Millipore) and blocked with 5% non-fat milk for 1 h at room temperature. Membranes were then incubated with primary antibodies (diluted 1∶1,000) including anti-rabbit p-AMPK, AMPK, p-ACC, ACC, PPARα, IRS-1, Glut-4 (plasma membrane protein), LXRα, LaminB1 and β-actin (Cell Signaling Technology, Inc., Massachusetts, USA) and anti-mouse adiponectin (Abcam) at 4°C overnight. After incubation with horseradish-peroxidase-conjugated goat anti-rabbit secondary antibodies at room temperature for 1 h, immunoreactive proteins were detected using a chemiluminescent ECL assay kit (Millipore).

### Culture and differentiation of 3T3-L1 cells

3T3-L1 cells were purchased from the American Type Culture Collection (ATCC) and were cultured in Dulbecco’s modified Eagle medium (DMEM) containing 10% fetal bovine serum (FBS) (Bio-rad, Hercules, CA, USA) and 100 IU/ml penicillin/streptomycin at 37°C in an atmosphere of 5% CO_2_ and 95% humidity. Two days post-confluent cells (designated as Day 0) were induced to differentiate into adipocytes by the addition of differentiation mixture with DMEM containing 10% FBS, 10 µg/mL insulin, 0.5 mM 3-isobutyl-1-methylxanthine (IBMX), and 1 µM dexamethasone. Two days later, culture medium was changed to DMEM supplemented with 10% FBS and 10 µg/mL insulin for 2 days. The medium was then replaced every other day with DMEM containing 10% FBS for different periods. On day 10, 3T3-L1 cells were treated with different concentrations of the LXR agonist T0901317 (0, 0.1, 1.0 and 10 µM) or GW3965 (0, 1.0 and 10 µM) for 24 h using equal volumes of the vehicle DMSO as controls. Cells were harvested for the isolation of total RNA and protein extraction.

### LXRα RNA interference

The LXRα-specific shRNA expressing pGFP-V-RS Vector (shRNA-LXRα) and the HuSH 29-mer Non-Effective Scrambled pGFP-V-RS Vector (shRNA-Control) were purchased from Origene Company (Rockville, MD, USA). For virus packaging, Phoenix 293 cells (1×10^7^) were grown to 90% confluence in 10 cm dishes in DMEM containing 25 mM glucose, 100 IU/ml penicillin, 100 µg/mL streptomycin and 10% FBS in an atmosphere of 5% CO_2_ at 37°C. The shRNA transfections into the packaging cells were performed using Lipofectamine 2000 reagent (Invitrogen) and the transfection efficiency was monitored by fluorescence microscopy. At 48 h after transfection, cell medium containing virus (retrovirus solution) was harvested, centrifuged and filtered (0.45 µm). Undifferentiated 3T3-L1 cells at 60–70% confluence were repeatedly treated for 48 h with a 1∶1 mixture of the retrovirus solution and culture medium with addition of polybrene at a final concentration of 8 µg/mL. Transfected 3T3-L1 cells were selected with medium containing 2.0 µg/mL puromycin (Sigma-Aldrich, Saint Louis, Missouri, USA) for 48 h to eliminate uninfected cells. Stably transfected 3T3-L1 cells were then induced to differentiate as described previously. The efficiency of interference of LXRα protein in 3T3-L1 cells was verified by qRT-PCR and Western blotting at 0, 5 and 10 days of the differentiation period. Total RNA and protein were extracted from differentiated cells at 10 days for qRT-PCR and Western blotting analyses.

### ChIP study

For chromatin immunoprecipitation (ChIP) experiments, chromatin was extracted from 3T3-L1 adipocytes on day 10 after differentiation using the Simple ChIP Kit (Cell Signaling Technology, Inc., Massachusetts, USA). Cells were treated with different concentrations of the LXR agonist T0901317 (0, 0.1, 1.0 and 10 µM) for 24 h. Equal volumes of the vehicle DMSO were used as the controls. Cells were then cross-linked for 10 minutes with 1% formaldehyde, then lysed and sonicated three times for 20 seconds using a sonic dismembrator. Lysates were precleared with protein A agarose beads. PPARγ antibody (Cell Signaling Technology, Inc., Massachusetts, USA) was applied. DNA was released from protein-DNA complexes by proteinase K digestion and then subjected to quantitative real-time PCR analysis of the adiponectin response elements for PPARγ (PPRE) using the SYBR Green qPCR Kit (TaKaRa) and the adiponectin PPRE primers: Forward: 5′-GGTGCTGGGAATTGAACTCA-3′; Reverse: 5′-CCTGTTTCCAGGCTTTGGCC-3′. Using a non-PPRE region of adiponectin promoter as a negative control with following primers: Forward: 5′-CTGAC GACAC CAAAA GGGCT C-3′; Reverse: 5′-TCCAA CCTGC ACAAG TTCCC-3′. ChIP-qPCR data were normalized to input samples for the amount of chromatin and for immunoprecipitation efficiency by normal IgG controls.

### Plasmids

The LXRα-specific shRNA expressing pGFP-V-RS Vector and HuSH 29-mer Non-Effective Scrambled pGFP-V-RS Vector were purchased from Origene Company (Rockville, MD, USA); (*pGL*3.0-PPRE-Luc, PPRE-Luc), a Renilla *pGL*4.75 [hRluc/CMV] (*phRL* CMV), *pcDNA* PPARγ, *pcDNA* LXRα and *pcDNA* 3.1(+) were kind gifts from Dr. Wuqiang Fan (University of California, San Diego, USA).

### Multiple plasmid transfections and Luc assays

HEK293 were grown at 37°C in an atmosphere of 5% CO_2_ in DMEM containing 25 mM glucose, 100 IU/mL penicillin, 100 µg/mL streptomycin and 10% FBS. Transfections of HEK293 were carried out in 12-well plates. The indicated amounts of each of the expression plasmids *pcDNA* PPARγ and *pcDNA* LXRα (0.2 µg each) were transfected simultaneously with a PPRE-Luc reporter plasmid (0.4 µg) and a *phRL* CMV plasmid (0.02 µg) with Lipofectamine 2000 (Invitrogen). Control transfections were performed with *pcDNA* 3.1 (0.4 µg) in HEK293 cells. At 24 h after transfection, cells were treated with 10 µM T0901317 or 3 µM Pioglitazone or together, or different doses of T0901317 in DMEM containing 10% FBS, and incubated for a further 24 h. Subsequently, the amount of Luc activity in transfectants was measured and normalized to the amount of Renilla luciferase activity using the Dual-Luciferase Reporter Assay System (Promega, Wisconsin, USA).

### Statistical analysis

Data are expressed as means ± SEM (*in vivo* studies) or means ± SD (*in vitro* studies). Differences between the means of individual groups were analyzed with independent *t*-tests or one-way ANOVA and LSD multiple range tests. Two-way repeated measures were used for comparisons between glucose and insulin levels of IPGTT or ITT using the statistical software package SPSS 16.0. A significant difference was defined as *P*<0.05. Each *in vitro* experiment was conducted in triplicate.

## Results

### T0901317 induced insulin resistance in C57BL/6 mice fed on regular chow diet

**Figure 1:**
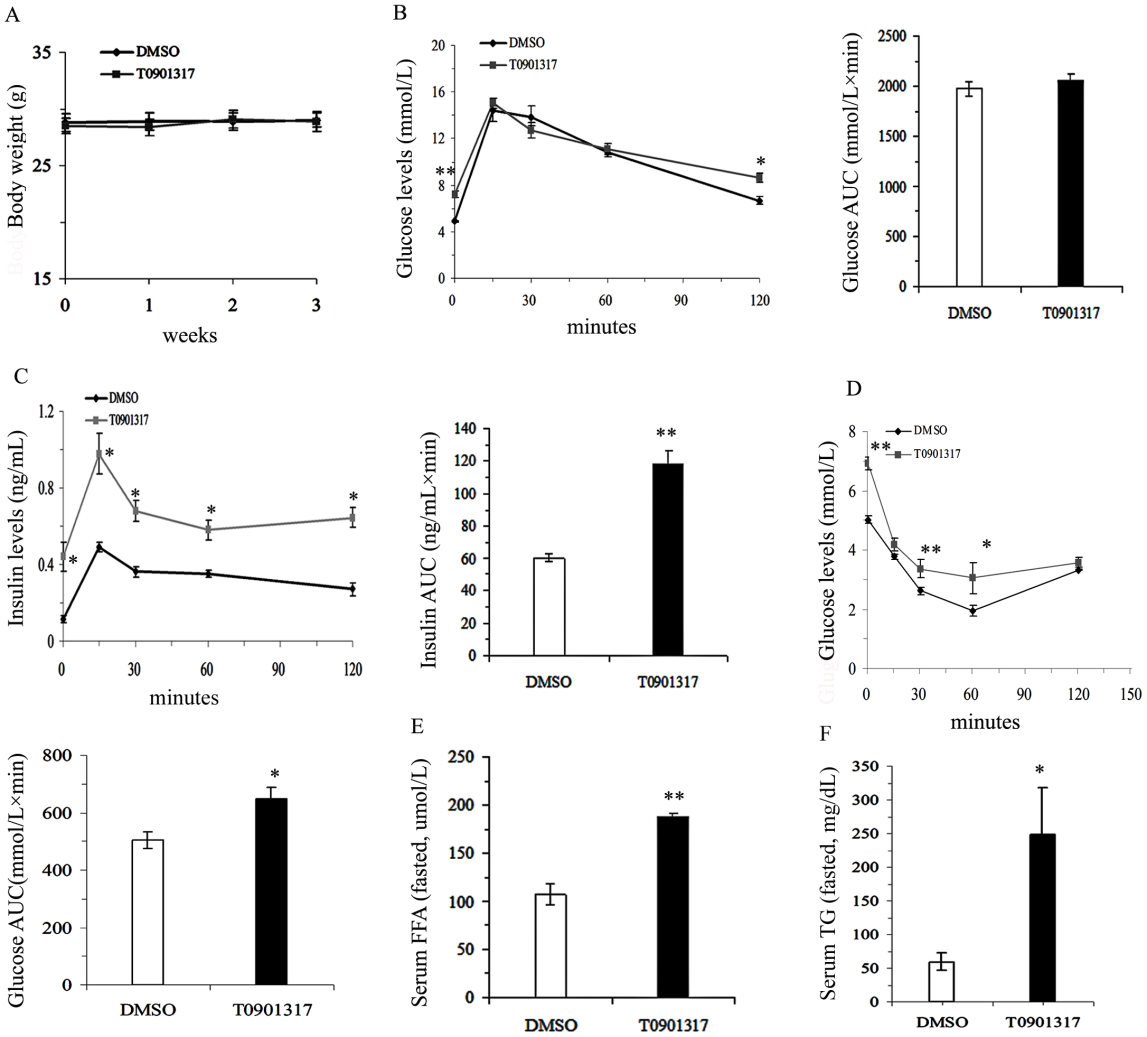
Effects of T0901317 on insulin function. A–F, Body weight (A, *n* = 14–16 mice per group), glucose levels and glucose level area under the curve (AUC) during an intraperitoneal glucose test (IPGTT, 1.5 g glucose per kg body weight) (B), insulin levels and insulin level area under the curve (AUC) during IPGTT (C, *n* = 7–8 mice per group), glucose levels and glucose level area under the curve (AUC) during an insulin tolerance test (ITT, 0.5 IU insulin per kg body weight, *n* = 7–8 mice per group) (D), free fatty acid levels (E) and triglyceride levels in serum after an overnight fast (*n* = 7–8 mice per group). All values are presented as mean ± SEM, *P<0.05 and **P<0.01 vs. control mice treated with DMSO.

As shown in Figure 1, treatment with the LXR agonist T0901317 (30 mg/kg. d i.p.) for 3 weeks had no effect on body weight at the end of treatment (*P*>0.05, Fig. 1A). Although both fasting and 120 min glucose levels of IPGTT were higher in T0901317-treated mice, similar area under the curve (AUC) values of the glucose levels during IPGTT were observed in the two groups (AUC Glu, *t* = −0.773, *P*>0.05, Fig. 1B). However, the insulin response at different time-points, as well as the AUC values during the IPGTT, were all significantly higher in T0901317-treated mice than those in DMSO-treated control mice (AUC Ins, *t* = −7.143, *P*<0.01, Fig. 1C), suggesting that LXR activation decreased insulin action. Decreased insulin sensitivity was further confirmed by ITT. T0901317-treated mice showed higher glucose levels at several time-points after insulin injection (i.p.), leading to a higher glucose AUC in T0901317-treated mice (AUC Glu, *t* = −3.131, *P*<0.05, Fig. 1D). FFA levels, a well established risk factor of insulin resistance, and TG levels were significantly higher in the T0901317-treated mice as compared to those in the control mice (FFA, *t* = −7.058, *P*<0.01, Fig. 1E; TG, *t* = −2.368, *P*<0.05, Fig. 1F).

### T0901317 decreased fat mass and circulating adiponectin levels in C57BL/6 mice

**Figure 2:**
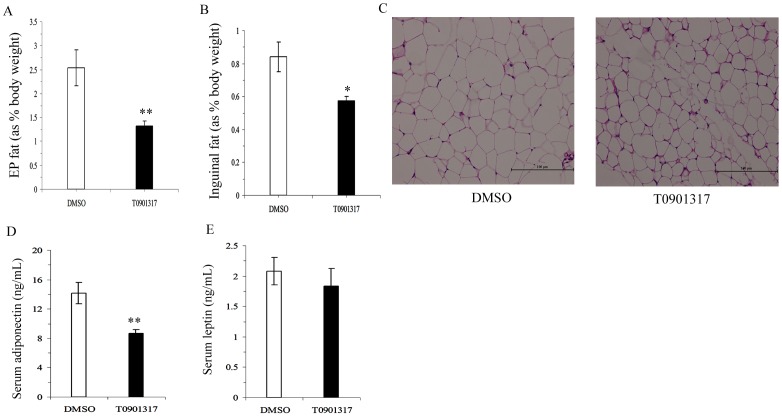
Effect of T0901317 on fat mass and adipokines. **A–E**, the percent of epididymal fat mass (A, % as body weight) and inguinal fat mass (B, % as body weight, *n* = 14–16 mice per group), H&E staining of EP fat (C, ×200 magnification), adiponectin levels in serum (D) and leptin levels in serum (E, *n* = 7–8 mice per group). All values are presented as mean ± SEM, *P<0.05 and **P<0.01 vs. control mice treated with DMSO.

Dysregulated adipose metabolism has been suggested to play an important role in insulin resistance. To explore its possible involvement in whole-body insulin sensitivity, we studied the changes in fat pad morphology and adipokine secretion in response to T0901317 treatment. Although no changes in body weight were observed following T0901317 treatment, the weights of both epididymal and inguinal fat pads as normalized to body weight were significantly reduced in T0901317 treated mice (EP fat %, *t = *3.428, *P*<0.01, Fig. 2A; Inguinal fat %, *t = *2.791, *P*<0.05, Fig. 2B). Furthermore, H&E staining showed that the size of adipose cells in the EP fat of T0901317 treated mice appeared smaller than those of the controls (Fig. 2C). In spite of the decreased fat mass, adiponectin levels in the circulation were significantly decreased in T0901317-treated mice as compared with those of the control mice (*t = *3.576, *P*<0.01, Fig. 2D), while there was no difference in the serum leptin levels between the two study groups (*t = *0.665, *P*>0.05, Fig. 2E).

### T0901317 decreased adiponectin transcription and its signaling in EP fat

**Figure 3:**
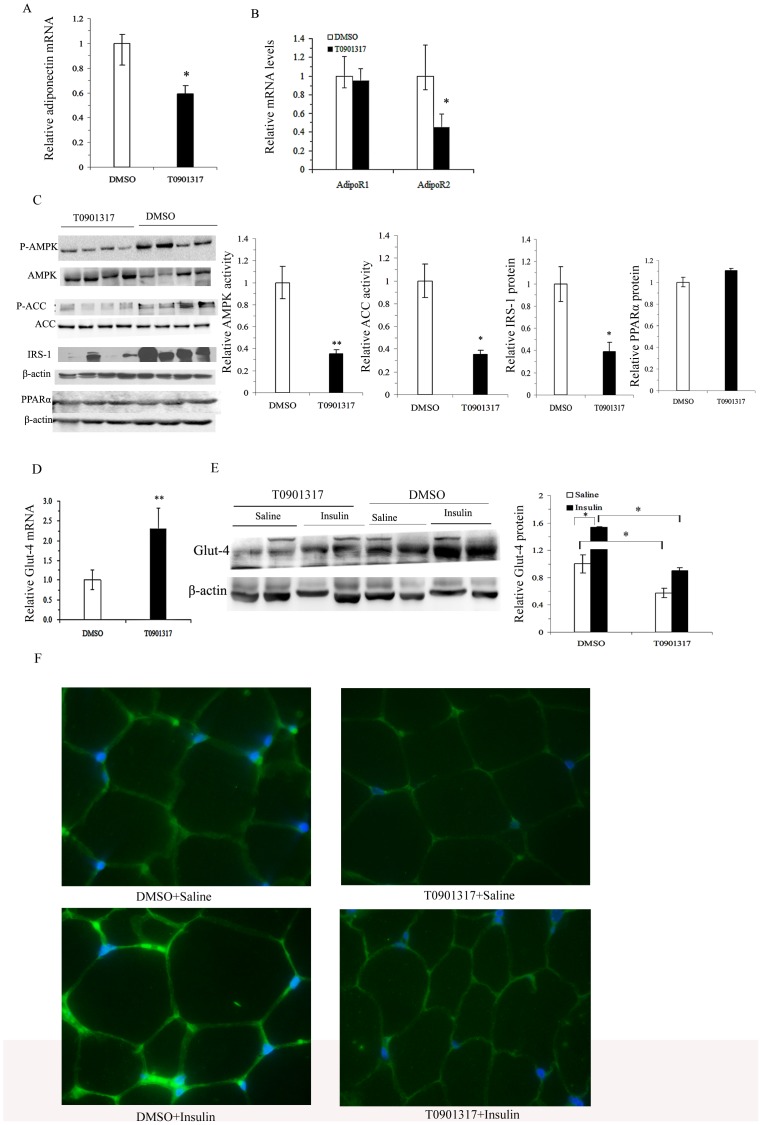
Effect of T0901317 on adiponectin signaling in EP fat. A–D, quantitative real-time RT-PCR analyses of adiponectin mRNA (A) and its receptors 1 (AdipoR1) and 2 (AdipoR2) mRNAs (B) in EP fat; Western blot analyses of phosphorylation of AMPK, phosphorylation of ACC, IRS-1 and PPARα protein levels using β-actin as a loading control (C); quantitative real-time RT-PCR analysis of Glut-4 mRNA in EP fat (D). All values are presented as means ± SEM, *n* = 4–6 mice per group. *P<0.05, **P<0.01 vs. control mice treated with DMSO. E–F, both basal and insulin-stimulated Glut-4 membrane translocations detected by Western blotting using β-actin as a loading control (E); and immunofluorescence detection of membrane Glut-4 (F, ×1000 magnification). Values are presented as means ± SEM, *n* = 4 mice per group injected i.p. with insulin or saline, *P<0.05 as indicated.

Adiponectin acts both locally and distally by autocrine, paracrine and endocrine mechanisms. Adipose tissue is not only the origin of adiponectin, but also a target of its function. We then tested the adiponectin signaling in EP fat. In accordance with the decreased serum adiponectin levels, the adiponectin mRNA levels in EP fat were also decreased in T0901317-treated mice compared to those in control mice (Fig. 3A).

Compared with the DMSO-treated controls, AdipoR2, but not AdipoR1 was downregulated in EP fat by T0901317 treatment (Fig. 3B). Phospho-AMPK levels, as well as phospho-ACC levels, the main downstream mediator of AMPK were significantly decreased in EP fat by T0901317 treatment (Fig. 3C). T0901317 treatment also significantly reduced the expression of the major insulin signaling protein, IRS-1, in EP fat (Fig. 3C). However, PPARα protein levels in EP fat were not changed by T0901317 treatment (Fig. 3C).

Glut-4 is the major glucose transporter responsible for glucose uptake in adipocytes and is also a critical downstream mediator of AMPK activity. We next assessed the effect of LXR activation on Glut-4 expression and membrane translocation. Although Glut-4 mRNA levels in EP fat were induced significantly by T0901317 treatment (Fig. 3D), translocation of Glut-4 to the membrane demonstrated by Western blotting (Fig. 3E) and IHC was significantly decreased by T0901317 treatment both at the basal level and after insulin stimulation (Fig. 3F).

### T0901317 had no effect on adiponectin signaling in the liver

**Figure 4:**
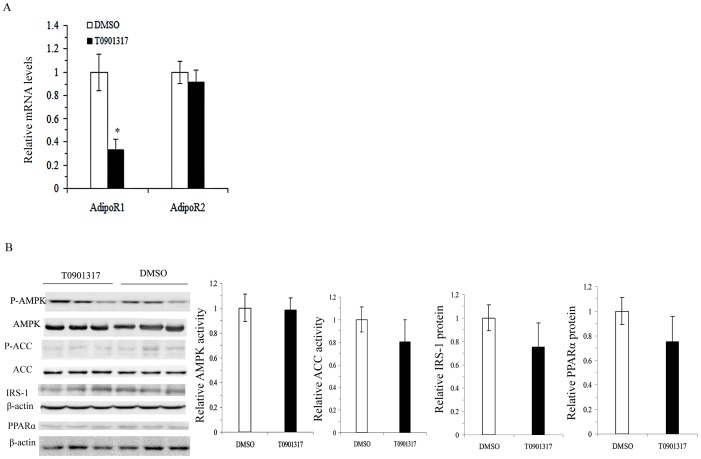
Effects of T0901317 adiponectin activity in liver. A–B, quantitative real-time RT-PCR analyses of adiponectin receptors 1 (AdipoR1) and 2 (AdipoR2) mRNAs in liver (A); Western blotting analyses of phosphorylation of AMPK, phosphorylation of ACC, IRS-1 and PPARα protein levels using β-actin as a loading control in liver (B). All values are presented as means ± SEM, *n* = 4–6 mice per group. *P<0.05, **P<0.01 vs. control mice treated with DMSO.

Liver is another important target of adiponectin activity; therefore, we investigated whether the decreased circulating adiponectin levels also resulted in changed adiponectin activity in the liver. As shown in Figure 4A, the expression of AdpoR1 in the liver was deceased following T0901317 treatment; however, the expression of AdipoR2, the dominant receptor mediating adiponectin activity in the liver, remained unchanged (Fig. 4A). Similarly, the post-receptor events of AMPK-ACC activity and PPARα levels, as well as IRS-1 levels were all unchanged in the liver of the T0901317 treated mice (Fig. 4B).

### Activation of LXR downregulated the expression of adiponectin possibly by interfering with PPARγ signaling in vitro

**Figure 5:**
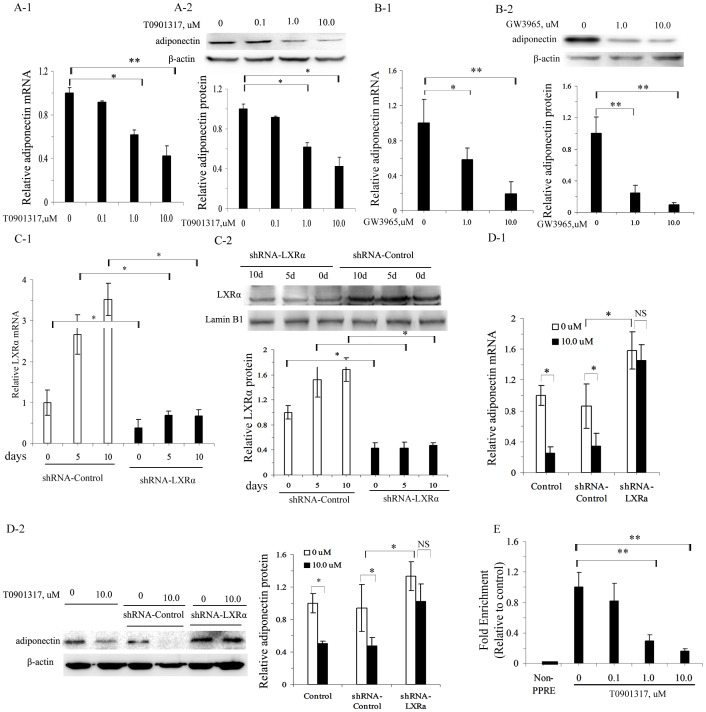
Effect of LXR activation on adiponectin expression *in* *vitro*. A–E, adiponectin mRNA by quantitative real-time RT-PCR (A-1) and adiponectin protein levels by Western blotting using β-actin as a loading control (A-2) in differentiated adipocytes treated with different doses of T0901317 for 24 h; analysis of adiponectin mRNA by quantitative real-time RT-PCR (B-1) and adiponectin protein levels by Western blotting using β-actin as a loading control (B-2) in differentiated adipocytes treated with different doses of GW3965 for 24 h; quantitative real-time RT-PCR analysis of LXRα mRNA (C-1) and Western blotting analysis of LXRα protein levels using LaminB1 as a loading control (C-2) in LXRα silenced 3T3-L1 adipocytes at 0, 5 and 10 days during the differentiation periods; quantitative real-time RT-PCR analysis of adiponectin mRNA (D-1) and Western blotting analysis of adiponectin protein using β-actin as a loading control (D-2) in differentiated adipocytes with LXRα silencing and treated with 10 µM T0901317; ChIP-qPCR analysis of the binding of PPARγ to adiponectin PPRE in differentiated adipocytes treated with different doses of T0901317 (E, data were normalized to input samples for the amount of chromatin). The results are presented as the mean ± SD of three replicates in three separate experiments.*P<0.05, **P<0.01, NS non-significant as indicated.

The effect of LXR activation on adiponectin expression was further studied *in vitro* using two synthetic LXR agonists, T0901317 and GW3965. Both T0901317 and GW3965 decreased adiponectin gene transcription and protein levels in 3T3-L1 mature adipocytes in a dose-dependent manner with a maximum effect at 10 µM (Fig. 5A-1, A-2; 5B-1, B-2).

The effect of T0901317 on adiponectin expression was at least partly mediated by LXRα. As shown in Figure 5C, retrovirus-mediated LXRα-knockdown in pre-adipocytes led to decreased LXRα mRNA and protein levels at 0, 5, and 10 days of the differentiation period (Fig. 5C-1, C-2). However, LXRα-silencing led to an increase in adiponectin expression at both the mRNA and protein levels compared to the levels in cells transfected with the shRNA-Control. Furthermore, LXRα-silencing diminished the inhibitory effect of T0901317 (10 µM) on adiponectin mRNA and protein levels in mature adipocytes (Fig. 5D-1, D-2). Adiponectin transcription is predominantly regulated by PPARγ; no functional LXREs have been identified in the promoters of the murine and human adiponectin genes as yet. Therefore, we investigated whether LXR activation interfered with the binding of PPARγ to PPRE in the adiponectin promoter by ChIP assay. qPCR products were found using a primer set spanning PPRE of adiponectin promoter, but none was detected with the negative control primers. Furthermore, the binding of PPARγ to the adiponectin PPRE was inhibited by T0901317 in a dose-dependent manner, with approximately 70% and 84% reduction in fold enrichment at concentrations of 1.0 and 10.0 µM, respectively (Fig. 5E).

### LXR activation downregulated PPRE-Luc activity induced by PPARγ in HEK293 cells

**Figure 6:**
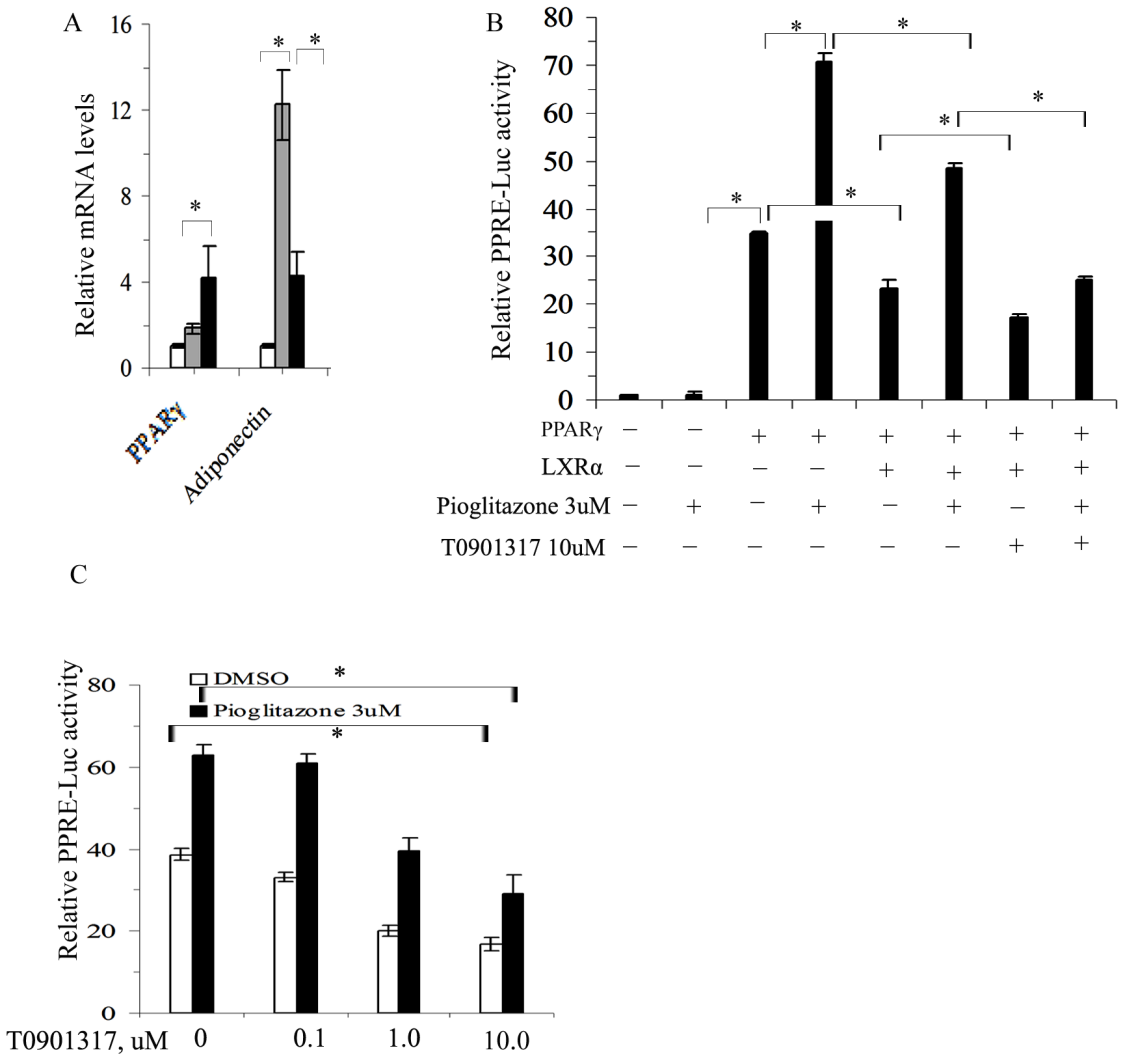
Effects of T0901317 on PPARγ transcriptional activity. A–C, quantitative real-time RT-PCR analyses of PPARγ and adiponectin expressions in differentiated adipocytes treated with 3 µM Pioglitazone in the presence or absence 10 µM T0901317 (A, white bar-DMSO treatment, grey bar-3 µM Pioglitazone treatment, black bar- 3 µM Pioglitazone and 10 µM T0901317 treatment); double promoter luciferase reporter assay of PPRE-Luc activity in HEK293 cells co-transfected with LXRα or PPARγ or both and treated with different drugs (B, cells subjected to the same basic co-transfection () with 0.4 µg PPRE-Luc +0.02 µg *phRL* CMV in each group); double promoter luciferase reporter assay of PPRE-Luc activity in HEK293 cells subjected to multiple plasmid co-transfections with (
+0.2 µg *pcDNA* PPARγ+0.2 µg *pcDNA* LXRα) and treated with different doses of T0901317 in the presence or absence of 3 µM Pioglitazone (C). The results are presented as the mean ± SD of three replicates in three separate experiments.*P<0.05 as indicated.

Adiponectin is a typical PPARγ transcriptionally regulated gene, and is primarily stimulated by PPARγ agonist. To clarify the effect of LXR activation on PPARγ transcriptional activity, we analyzed the expression of PPARγ and adiponectin in presence of a PPARγ agonist Pioglitazone. As expected, Pioglitazone (3 µM) significantly induced the expression of adiponectin, whereas it only slightly upregulated PPARγ expression. However, the increase in adiponectin induced by Pioglitazone was largely diminished by presence of 10 µM T0901317. In contrast, the expression of PPARγ was increased under 3 µM Pioglitazone together with 10 µM T0901317 (Fig. 6A).

To further elucidate the effects of LXR activation on PPARγ transcriptional activity, transfections studies were performed with HEK293 cells using the firefly luciferase reporter gene assays containing a PPRE from the mouse *Gpd1* promoter (pPPRE-Luc) as a representative PPARγ target. We confirmed that PPRE-Luc activity was markedly (35-fold) induced by co-transfection of the PPARγ plasmid due to lack of endogenous PPARs expression in HEK293 cells. The PPRE-Luc activity was further increased by the addition of 3 µM Pioglitazone. However, the PPARγ-inducible Luc activity was decreased by co-transfection of the LXRα plasmid regardless of the presence of Pioglitazone. Addition of 10 µM T0901317 augmented the inhibitory effect of LXRα on PPRE-Luc activity with or without co-treatment with 3 µM Pioglitazone (Fig. 6B). The inhibitory effect of LXR activation on PPRE-Luc activity was further evaluated in terms of dose-dependency. As shown in Figure 6C, T0901317 inhibited the PPARγ activation of PPRE-Luc in a dose-dependent manner with very similar inhibitory efficiency with or without 3 µM Pioglitazone (Fig. 6C).

## Discussion

LXR has been recognized not only a key regulator of cholesterol metabolism, but also a suppressor of inflammatory signaling in macrophages; thus it has been identified as a promising pharmacological target for the management of atherosclerosis [2], [23]. For decades it has been known that diabetes and atherosclerosis are closely associated and often develop in parallel [24]. Inconsistencies in reports of the effects of LXR activation on whole-body insulin sensitivity has raised concerns about the possibility of increased diabetes associated with as an anti-atherosclerosis target [6]–[9]. In the current study, we discovered a potential mechanism by which LXR activation induced insulin resistance based on dysregulated adiponectin activity following T0901317 treatment.

Consistent with the results of Archer et al. [25], we also found that LXR activation had no effect on body weight change but decreased visceral fat mass. However, in contrast to Archer’s study, which was increased, we also found a decline in the percentage of subcutaneous fat. This difference might be explained by the choice of different sources of representative subcutaneous fat, as well as differences in mice models selected and relatively smaller dose (GW3965 10 mg/kg) adopted in their study. Decrease in fat mass and adipocyte size caused by LXR activation was also reported in other studies both *in vivo* and *in vitro* studies through increasing oxygen consumption rate and lipolysis [25]–[28]. PPARγ has been identified as the central regulator of adipocyte biology since 1994, convincing evidence for a critical role of PPARγ in adipogenesis was mainly from experiments in which PPARγ null mouse embryonic fibroblasts can not undergo adipogenesis *in vitro* and fat-specific PPARγ knockout mice (PPARγ FKO) exhibited fat pad loss, decrease in adipokine secretion and insulin sensitivity [29]–[31]. The antagonistic effect of chronic LXR activation on PPARγ’s transcriptional activity in adipocyte found in our present study might also be a possible mechanism mediating the fat mass decrease.

Despite the absence of changes in body weight, insulin action was decreased by LXR activation as shown by insulin overproduction during IPGTT and higher glucose AUC during ITT. However, the response of T0901317 treated mice to glucose challenge in the IPGTT assay was not as significant as that in ITT, although the blood glucose levels at 0 and 120 min were higher than those of DMSO-treated control mice. It can be speculated that this is caused by the compensatory insulin secretion, because insulin levels of T0901317-treated mice were significantly higher than those of the controls. The discrepancy between our results and those of others showing improved insulin sensitivity following LXR activation may be attributed to the different animal models employed; one employing an animal model fed on a regular chow and the others using a model of diet-induced or genetic obesity that has already developed insulin resistance [6]–[7], [25], [27], [32]. Evidence supporting this hypothesis is provided by Grefhorst et al [32] showing improved insulin sensitivity by LXR activation in ob/ob mice but non-changed hepatic and whole-body insulin sensitivity in lean mice fed on a regular chow diet. However, they did not show LXR activation resulted in insulin resistance in lean mice. This discrepancy could be partly explained by relatively longer treatment period of our study than theirs (21d vs 10d), since we observed a significant smaller fat mass by 3 weeks of T0901317 treatment, which possibly contributed to a decrease in adipokine (i.e adiponectin) level as shown in our study.

The vast majority of studies concerning the role of LXR activation in metabolic pathways were performed in non-adipose cells/tissues, and the contribution of LXR activation to insulin function in adipose tissue is unclear. Adiponectin deficiency is closely related to insulin resistance. Adiponectin is also known as a critical mediator of PPARγ-agonist-mediated improvements in insulin sensitivity [33]. In contrast to the effects of PPARγ activation, our present study clearly showed that LXR activation significantly decreased the transcription of adiponectin in EP fat, resulting in decreased adiponectin levels in the circulation. The results of our *in* *vivo* study were further confirmed *in vitro.* LXRα-silencing diminished the effect of T0901317 on adiponectin expression suggesting a dominant role of the LXRα isoform in this process. Previous studies using human Chub-S7 adipocytes demonstrated only slight upregulation of adiponectin mRNA in response to T0901317 treatment, although this effect was not observed in the SVF of human adipose tissue. This discrepancy might be explained by the different cell models adopted and different experimental designs [34].

Adiponectin acts locally and distally after secretion and adipose tissue is also an important target of the autocrine function of adiponectin. AdipoR2 and AdipoR1 are abundant in liver and muscle, respectively, with neither receptor having been shown to predominate in mediating the activity of adiponectin in fat pads to date [35]. Our current study demonstrated that LXR activation decreases expression of AdipoR2 but not AdipoR1, with deceased activity of AMPK activity and its downstream mediators of ACC in EP fat, thus confirming a decrease in adiponectin activity in EP fat. Results from Kudoh et al. [36] also suggested a relatively more important role of AdipoR2 in mediating adiponectin activity in 3T3-L1 adipocytes following PPARγ stimulation.

Glut-4 is mainly responsible for basal and insulin-stimulated glucose uptake in fat tissue. In accordance with other studies [27], [37]–[38], we also found that LXR activation induced Glut-4 gene expression. However, basal and insulin-stimulated Glut-4 membrane translocation was significantly decreased, which may be associated with decreased glucose utilization in EP fat. Consistent with our findings, a recent report by Pettersson et al. [11] also described impaired Glut-4 protein translocation in 3T3-L1 adipocyte following T0901317 treatment. It can be hypothesized that decreased Glut-4 membrane translocation is due to decreased AMPK activity based on studies that have revealed that WAT and skeletal muscle regulation of glucose uptake are distinctly affected by AMPK activation [39]–[40].

It is unlikely that LXR activation directly inhibits the transcription of adiponectin due to the deficiency of LXRE in its promoter. The transcription of adiponectin is dominantly regulated by PPARγ and significantly induced by PPARγ agonist. In the present study, ChIP analysis revealed that LXR activation inhibited the binding of PPARγ to adiponectin PPRE. Furthermore, LXR activation exhibited the antagonistic effect on adiponectin transcription even under the presence of PPARγ agonist Pioglitazone. The antagonistic effect of LXR activation on the PPARγ transcriptional activity was further supported by the observation of suppressed PPRE-activity in HEK 293 cells following LXRα co-transfection and T0901317 treatment in or without presence of Pioglitazone. In contrast, the presence of LXRE in the promoter of PPARγ makes it unsurprising that its expression in EP fat was increased by LXR activation [14]. However, increased PPARγ expression did not result in upregulation of adiponectin, suggesting an involvement of other mechanisms mediating the antagonistic effect of LXR activation on PPARγ signaling.

The cross-talk between LXRs and PPARα nuclear receptors has long been recognized in liver.[21], [41]–[42] However, this is the first report revealing the possible existence of cross-talk between LXRs and PPARγ in adipose tissue. It has been shown that LXR activation decreased PPARα signaling by competition for the common RXR receptor with PPARα and by directly binding to PPARα protein in hepatocytes [21], [41]. Whether the relative shortage of RXR and the existence of competition for the common receptor are also true in adipocytes requires further investigation. It is worth pointing out that the antagonistic effect of chronic LXR activation on PPARγ transcriptional activity might result in insulin insensitivity regarding to the dominant role of PPARγ in regulation of insulin-sensitizing adipokine (i.e adiponectin) secretion in adipose tissues.

In summary, in present study we provide evidence that LXR activation induces insulin resistance in C57BL/6 mice fed a regular chow diet. Furthermore, our data indicate that this might be associated with decreased adiponectin signaling in EP fat. The inhibitory effect of T0901317 on adiponectin expression highlights cross-talk between LXR and PPARγ signaling in adipose tissue. These findings raise the concern that LXR activation might increase insulin resistance-related disease and that this should emphasized when considering LXRs as a therapeutic target for atherosclerosis, especially in non-insulin-resistant individuals.
